# Automated low-cost framework for crack measurements in RC structures using deep learning approach

**DOI:** 10.1038/s41598-026-50880-w

**Published:** 2026-05-08

**Authors:** Mahmoud Hassouna, Mohamed Marzouk, Eissa Fathalla

**Affiliations:** Structural Engineering Department, Faculty of Engineering, Cairo University, Giza, 12613 Egypt

**Keywords:** Reinforced concrete cracks, Crack detection, Deep learning, Image calibration, Engineering, Materials science, Mathematics and computing

## Abstract

This research presents a comprehensive and automated framework for detecting surface cracks and measuring their widths in reinforced concrete (RC) members using a modified YOLO-V11 deep learning (DL) architecture. Basically, manual surface-crack inspection is subjective and labor-intensive, relying heavily on an inspector’s skill and conditions on-site. This often leads to inconsistent assessments and longer inspection time. Thus, the proposed approach mitigates these limitations by integrating automated crack detection with direct quantitative crack measurement. The proposed framework presents: (1) a DL crack segmentation model trained on a diverse dataset to enhance generalization in realistic inspection conditions for crack detection and segmentation, (2) a crack width measurement algorithm using patching and stitching method, and (3) a customized image calibration and scaling approach to transfer crack dimensions from pixel-size to real-size using low-cost imaging devices and tools. Finally, the proposed framework was validated using 230 measured crack points collected from both experimental specimens and existing RC structures. The prediction accuracy reached a coefficient of variation of 16.82% and a mean relative error of 12.65%, confirming the reliability of the proposed framework for crack measurements of RC structures.

## Introduction

Existing reinforced concrete (RC) cracks speed up the ingress of harmful environmental components such as chlorides, CO_2_, and moisture into concrete, reaching the steel reinforcement, which results in initiating and accelerating the corrosion process of steel reinforcement^1^. The corrosion of the steel reinforcement affects negatively the bond between the steel and the concrete^2^, in addition to the loss of the cross-sectional area of the steel rebar^3^. Thus, monitoring cracks is crucial for maintaining the safety of RC structures and prolonging their service life. From an assessment point of view, cracks are key indicators for the deterioration of RC structures. The magnitude and the location of the cracks of RC structures are linked to their remaining service life^4,5^. Fathalla et al.^6^ introduced a relationship between the remaining fatigue life of in-service RC bridge decks and site-inspected crack patterns. Mihaylov et al.^7^ estimated the residual life capacity of deep beams based on a single crack opening measurement. Liu et al.^8^ introduced a relationship between concrete cracks and loading ratio between applied load and maximum load capacity for concrete components using linear regression correlation. Hassan et al.^9^ studied how the crack width accelerates the initiation of the corrosion process using a probabilistic modelling framework. These studies demonstrate the importance of accurate on-site crack width measurement, which will be the main pivot for decision-making and maintenance activities. Moreover, given the high labor cost and the slow operation of manual crack measurements, this process needs to be automated^10^. Here, image-based crack detection has become a significant topic in structural health monitoring and asset management^11^. Studies clarified that image-based detection and automated evaluation of cracks can facilitate inspection coverage and repeatability while lowering inspection cost^12,13^. However, challenges still exist in terms of false positive and/or false negative results due to the machine model accuracy^14^.

In the past few decades, the research field has progressively adopted deep-learning models for quantitative crack detection^15^. Orinaitė et al.^16^ developed a machine-learning approach for detecting cracks in underwater concrete. Chun et al.^17^ introduced an automatic crack detection method using image-derived techniques combined with machine learning algorithms. Aravind et al.^18^ presented machine learning algorithms to detect cracks using image processing and failure pattern recognition techniques. Furthermore, numerous studies have demonstrated the effectiveness of the U-Net architecture and its various variants^19–22^. U-Net architecture has become a backbone for pixel-level crack detection and segmentation due to its high ability to preserve spatial detail, which is needed to resolve narrow cracks^23^. More recently, many researchers have moved to more real-time, multi-task detection and segmentation models (e.g., the YOLO family). The YOLO (You Only Look Once) family was first introduced in 2016^24^, presenting a revolution in computer vision by framing detection using a single regression problem. Recent works demonstrated that specialized YOLO variants have superiority over earlier segmentation architectures in crack detection, while offering much higher inference speed, especially YOLO-V11^25,26^. The YOLO family runs the entire image into the neural network in one forward path, which allows real-time processing with relatively high speed. YOLO family architecture can be further customized to enable more accurate results^27^. Moreover, it is feasible for deployment in drones, climbing robots, and inspection vehicles due to its high inference speed^28^. On the other hand, challenges emerge within this model when detecting very fine objects (e.g., thin cracks) since it requires high computational cost^29^.

The most important aspect in crack detection is to measure the crack width in pixels, which can be achieved by implementing several algorithms (e.g., Euclidean Distance Transform (EDT)^30^, Branch-growing (BG)^31^). Carrasco et al.^32^ used EDT. Zhang et al.^31^ used BG by deriving cracks graph (branches), then computing crack width using local closest-edge distances or optimized branch growth. Jakubowski and Tomczak proposed another approach using a deep learning meta-sensor that learns to infer width from high-resolution brightness profiles and local patches^33^. The main challenge in these methods is their high dependence on the quality of segmentation masks and skeleton pre-processing^34^.

The ultimate goal of crack detection is to measure the crack width in real size (e.g., in mm). In order to transfer the pixel size to real-size, different techniques can be used as follows:

1. Manual calibration: This method relies on introducing physical objects with known dimensions into the captured field of view. A common approach is to place a physical scale, marker, or checkerboard within the camera’s field of view. The scale factor can be obtained by measuring the number of pixels corresponding to a known physical length of the object used. Liu et al.^35^ used specimens marked by horizontal lines with 30 mm spacing to determine the crack width.
2. Laser projection calibration^36^: The laser projector is mounted on a camera so that the known geometry of the projected laser and the camera imaging model can be used to compute real-world distances from image measurements, either using laser triangulation/structured-light to recover 3-D coordinates of the surface or using a simpler scale calibration when the surface is planar. Nyathi et al.^37–39^ proposed a low-cost circular laser projection technique that creates a calibration marker in the image without physically placing a scale on the targeted surface. This study converted pixel widths to millimeters by detecting the geometry of the projected circular laser mark. Recent handheld systems using parallel laser-line and camera arrangements report high accuracy for thin cracks^40^.
3. Image–point cloud (LiDAR) fusion and photogrammetry^41,42^: Transferring pixel measurements into metric units is conducted by establishing a geometric one-to-one mapping between image rays and a metric 3D model (a LiDAR point cloud). Once the image is registered to the point cloud, each pixel can be projected into a 3D mesh, which can be used to measure real distances. Moreover, Multi-view Structure from Motion (SfM) and Multi-View-Stereo (MVS) frameworks^43^ generate metric 3D meshes in a similar way. Segmented edges reprojected onto these meshes allow true Euclidean width estimates that consider perspective and surface curvature^44^. Ozturk’s photogrammetric framework^45^ was implemented in drone mapping, and it achieved reliable millimeter-level estimates, given that images overlap and ground sampling distance (GSD) are sufficient. Other studies used stereo vision and photogrammetric techniques^46^ that recover local surface geometry and allow metric width estimation. The drawback of this method is its high acquisition cost and processing complexity.
4. Image–point cloud photogrammetry with rangefinder^47,48^: Mounting a rangefinder sensor on the camera and perform extrinsic calibration so that the range measurements are mapped into the camera coordinate system. Knowing camera intrinsics, the estimated range converts the angular pixel coordinates around that spot into metric offsets, producing a local mm/pixel scale. Kao et al.^49^ mounted four laser-ranging modules around the inspection camera to measure the object distance and finally calculate the metric image scale for each capture. Kim et al.^50^ used an ultrasonic distance sensor and a Wi-Fi module synchronized with a hybrid image binarization so that each image has an associated working distance. Then, the system uses this distance, camera geometry, and hybrid image processing to convert pixel measurements into actual crack widths. Ding et al.^51^ established a full-field scale calibration for the camera to calculate the mm/pixel scale by obtaining the benchmarks of the image-scale field under varying distances and angles using a digital image processing (DIP) and moving least squares (MLS) algorithm.

In the past years, researchers proposed a full package for crack detection and segmentation, including width measurement. Liu et al.^35^ proposed a DL model for crack detection and width measurement. They used a drawn scale on the specimen together with orthogonal captured photos to convert pixel-size to real-size. According to their results, they achieved good predictions for crack width with a relative error (RE) of 3.37–5.24%. Likewise, Xu et al.^52^ implemented a similar concept in measuring crack width, achieving predictions with a mean relative error (MRE) of 4.88%. Nyathi et al.^38,39^ used laser projection calibration to get the scale factor, and they achieved RE of 1–25%. On the other hand, automated calibration strategies using drones have been proposed by Ding et al.^51^ and Chou et al.^53^. They utilized drone-based imaging for calibration, achieving an MRE of 4.15% and 7.61%, respectively. Yan et al.^54^ proposed an automated approach using a drone mounted with three-dimensional LiDAR and achieved high accuracy with a RE of 10%.

Although these methods can achieve reliable accuracy, they encounter several practical challenges. In the case of manual calibration based on orthogonally captured photographs, it is hard to secure true orthogonal images of surfaces on-site since it depends heavily on the operator’s precision and often requires specialized tools. For drone-based imaging calibration, the primary limitation is accessibility, particularly during indoor inspections or when examining structures such as bridges over highways. Additionally, drones generally cannot approach surfaces closely due to safety constraints, increasing the need for high-resolution images. Thus, these issues limit the widespread adoption of drones for inspecting RC structures.

To advance a practical and cost-effective approach that leverages mobile device cameras, this paper presents a comprehensive framework for crack detection and width measurement using photographs captured by smartphones. The framework includes three key components: (1) a DL crack detection and segmentation model developed using a diverse dataset containing thousands of concrete crack images, (2) a segmentation procedure employing a patching and stitching technique to accurately isolate crack regions, (3) an image calibration and scaling process utilizing checkerboards to enable reliable crack width estimation. The framework is validated through crack width measurements obtained from large-scale experimental specimens and from crack images collected in existing RC structures. The validation results confirm the reliability of the proposed approach, demonstrating its potential as a cost-effective solution for crack measurements in RC structures.

## Methodology

The methodology implemented in this research represents a complete framework for automated crack segmentation and crack width measurement on concrete surfaces using a modern detector-based architecture (YOLO-V11)^55^. Ultimately, a software with a graphical user interface has been developed for crack detection and width measurement.

**Fig. 1:**
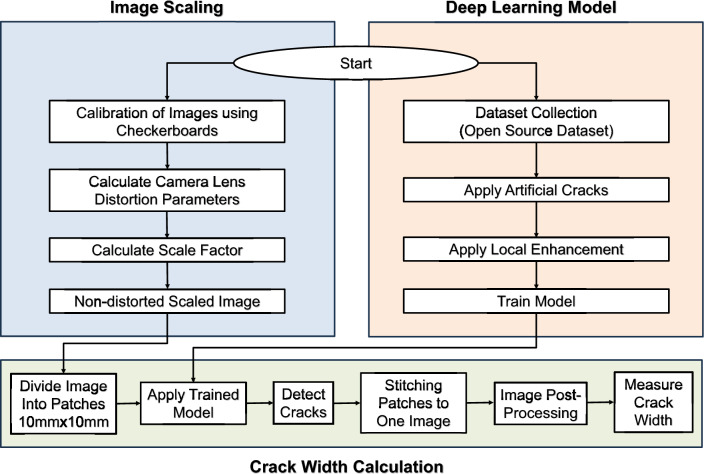
Proposed framework of crack detection.

The methodology consists mainly of three phases—see Fig. 1. These phases are summarized as follows:

- Developing a crack detection and segmentation DL model using collected dataset of concrete cracks.
- Image calibration and scaling to transfer from pixel-size to real-size (metric): a single-image calibration is conducted using four checkerboards placed at the corners of the image. This process includes the detection of the four checkerboards, local mm/pixel field estimation, perspective compensation, and uncertainty estimation.
- Crack width measurement using multiple methods: patching and stitching, crack skeletonization, and mask edge refinement.

### Dataset for crack segmentation model

YOLO-V11 architecture was selected for training the DL model since it delivers reliable detection performance (crack segmentation) while remaining deployable on regular platforms. Moreover, to perform crack width measurements, crack edges need to be detected accurately. Thus, the DL model was adapted by means of patching and stitching techniques in order to produce high-quality masks that can be suitable for metric measurements.

**Fig. 2:**
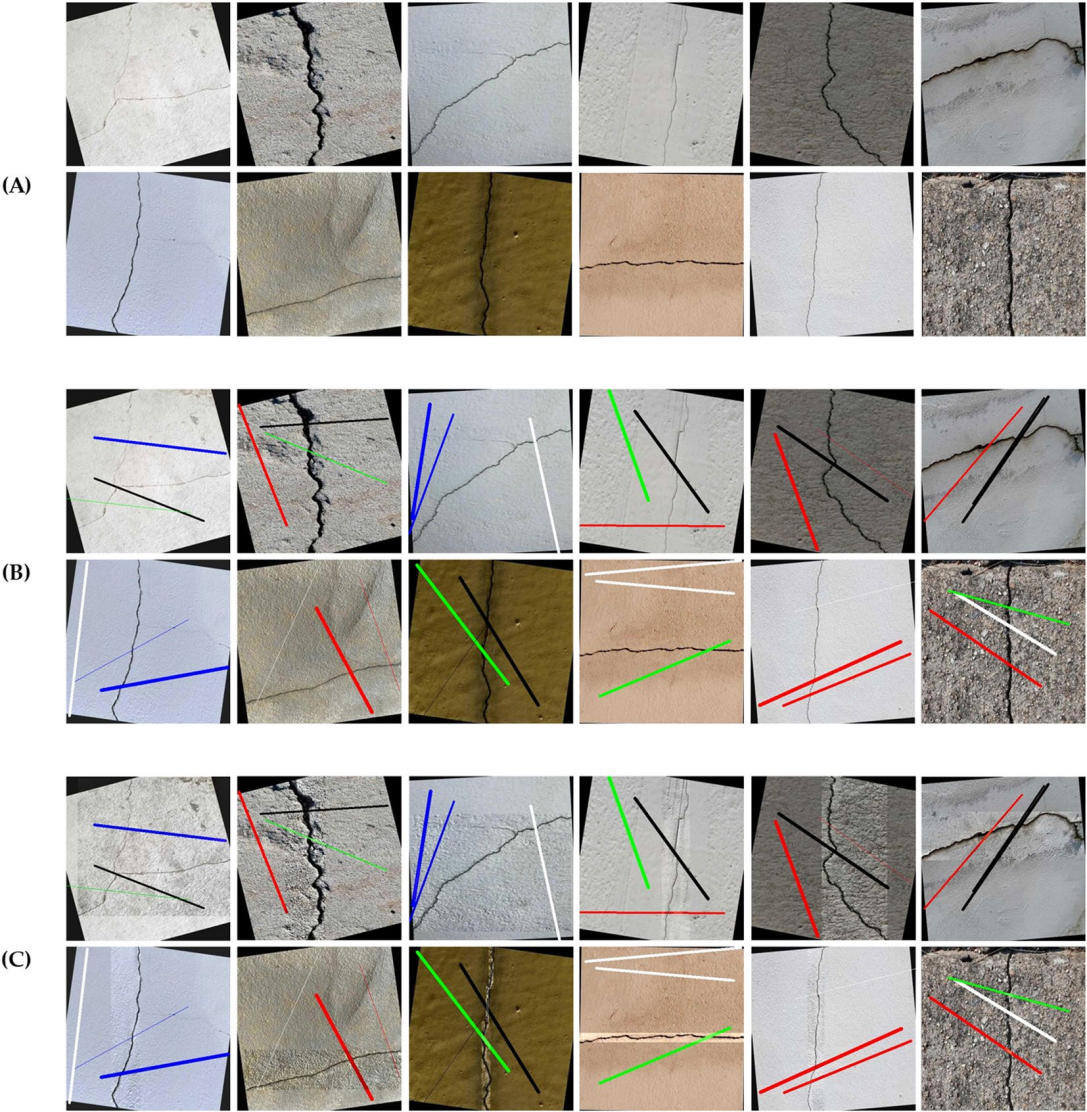
Samples of training dataset. (**A**) Raw images, (**B**) After adding artificial lines, and (**C**) After applying local enhancements

In order to build the DL model, a dataset is collected from online sources^56^. The dataset, namely, “dataset 1” comprises 4027 annotated images with a resolution of 416 × 416, focusing on concrete cracks. The imagery captures a diverse typology of concrete surfaces under varying loading and environmental conditions to facilitate the development of reliable object detection models. The primary focus is the identification and classification of all cracks in varying widths, lengths, and angles. These cracks are the result of environmental degradation and/or loaded experimental specimens, providing a ground-truth representation of structural deterioration. Each image is labeled with pixel-level masks to denote crack morphology. Samples of the dataset are shown in Fig. 2a, which includes a large variety of images of concrete cracks. At first, this dataset was used to train and build a preliminary DL model. The results of the preliminary DL model were promising; however, several false-positive predictions were observed in cases where images contained markers, painting lines, etc., due to periodic maintenance or other reasons. Here, it was decided to update dataset 1 by including artificial lines (i.e., colored lines with different thicknesses) on top of the original dataset. The new modified dataset, namely, “dataset 2” was randomly generated, considering the following random parameters of artificial lines: color, thickness, angle, and length—see Fig. 2b. Dataset 2 was divided into three subsets: training (70% of the dataset, 2818 images), validation (20% of the dataset, 806 images), and test subset (10% of the dataset, 403 images).

To further extend the training subset to generalize the model, augmentation was also applied (i.e., blurring, random noise, saturation), such that the training subset increased from 2818 to 8454 images, resulting in “dataset 3” of 9663 images. Next, local enhancement (i.e., sharpness and contrast) was applied to dataset 3, forming “dataset 4” of 19,326 images—see Fig. 2c. Ultimately, the final dataset “dataset 4” included a training subset (88% of the dataset), a validation subset (8% of the dataset), and a test dataset (4% of the dataset). The training of the DL model was conducted, using the YOLO-V11 algorithm on the Kaggle online platform for 60 epochs^57^.

## Image calibration and scaling

### Image calibration

Camera calibration is the process of estimating the parameters that map 3D world points into 2D image pixels. These parameters are divided into intrinsic and extrinsic parameters, which are used in undistorting the captured images. In this study, calibration of the imaging system has been performed using 4 planar checkerboards and a standard homography method^58^ using an OpenCV^59^ library on Python. The size of the checkerboards is 4 × 4 squares with a dimension of 10 mm. The checkerboards are placed at the corners of the field of view of the targeted object (crack). For each detected checkerboard, the corner coordinates of its squares are extracted and used to determine the distances between neighboring corners. This data is used to calculate the intrinsic parameters (e.g., the camera’s lens distortion coefficients) and extrinsic parameters (rotation and translation vectors), which are used to undistort the image using the standard pinhole camera model^58,60^.

### Homography mapping

After image calibration, intrinsic and extrinsic parameters are used to get the homography mapping. Homography is a concept of computer vision that defines the mathematical relationship between multiple images of the same planar surface in 3D space, using a pinhole camera model. In this study, instead of using multiple images, the four detected checkerboards, captured in a single photo, are used together with the calibration parameters. For each detected calibration checkerboard, the imaging relation reduces to a planar homography since all points on the checkerboard satisfy *Z* = *0*. *X*_*p*_ denotes a point on the checkerboard plane, and *x* denotes its homogeneous image coordinate. The relation of *x* and *X*_*p*_ is derived by a 3 × 3 homography *H* according to Eq. (1) to (3):

1 $$sx\, = \,H~X_{p}$$

2 $${X}_{p}={\left[\begin{array}{ccc}X& Y& 1\end{array}\right]}^{\top }$$

3 $$x={\left[\begin{array}{ccc}u& v& 1\end{array}\right]}^{\top }$$

where *s* is the projective scale factor.

Next, using the calibrated pinhole model, the homography is expressed by Eq. (4).

4 $$H=K\left[\begin{array}{ccc}{r}_{1}& {r}_{2}& t\end{array}\right]$$

where, *K* is the intrinsic camera parameters, *r*_*1*_ and *r*_*2*_ are the first two columns of the rotation matrix describing the checkerboard’s pose, *t* is the translation of the checkerboard origin in the camera coordinate system, and the third rotation column *r*_3_ is recovered as *r*_*3*_ = *r*_*1*_ × *r*_*2*_ when enforcing orthogonality.

The homography *H* is estimated from a minimum of four non-collinear point correspondences using the Direct Linear Transform (DLT)^61^ and subsequently refined through nonlinear optimization. To enhance robustness against possible mismatches, a robust estimator such as RANSAC is employed to identify a consistent set of inlier correspondences prior to refinement. By knowing the camera’s intrinsic matrix *K* from the calibration step, each homography *H* can be decomposed to obtain the checkerboard’s extrinsic parameters as in Eq. (5). This decomposition provides a metric estimate of the plane’s pose relative to the camera^58,62^.

5 $$\left[\begin{array}{ccc}{r}_{1}& {r}_{2}& t\end{array}\right]=\lambda {K}^{-1}H$$

where $\uplambda$ is a scalar normalization factor, and it is chosen such that it fulfils Eqs. (6) and (7).

6 $$\Vert {r}_{1}\Vert =\Vert {r}_{2}\Vert =1$$

7 $$r_{1}^{{\mathrm{T}}} r_{2} = 0$$

### Perspective correction and scale estimation

**Fig. 3:**
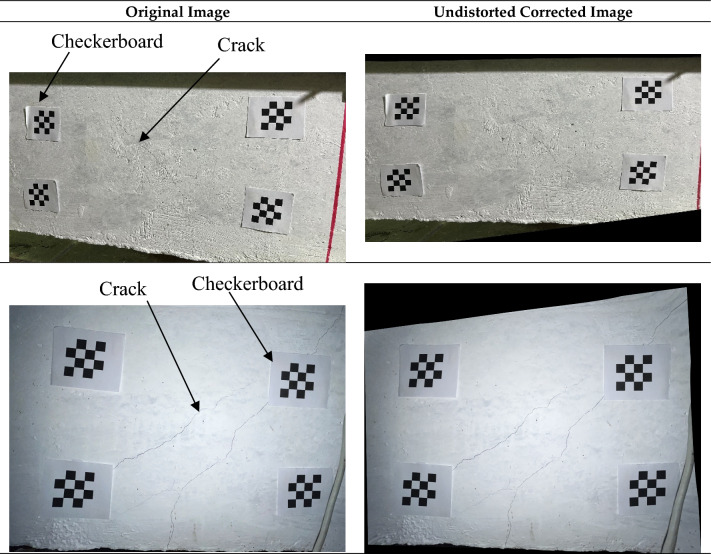
Process of camera calibration and perspective correction.

Given an estimated homography *H*, perspective correction to a desired metric is achieved by applying the inverse homography ${H }^{-1}$ to the image. The inverse mapping transforms the plane coordinates ${X}_{p}$ for each pixel’s center to the sampled image coordinates $x=H{X}_{p}$, which is used to produce the orthographic view. To convert between pixels and physical units, the scale factor $\alpha$ (mm/pixel) is computed in Eq. (8). Figure 3 shows examples of the images before and after perspective corrections and calibration.

8 $$\alpha = \frac{S}{{d_{pixel} }}$$

where, S is the checkerboard square’s dimension, and *d*_*pixel*_ is the measured distance between adjacent internal corner points on the rectified plane.

### Crack width measurement

**Fig. 4:**
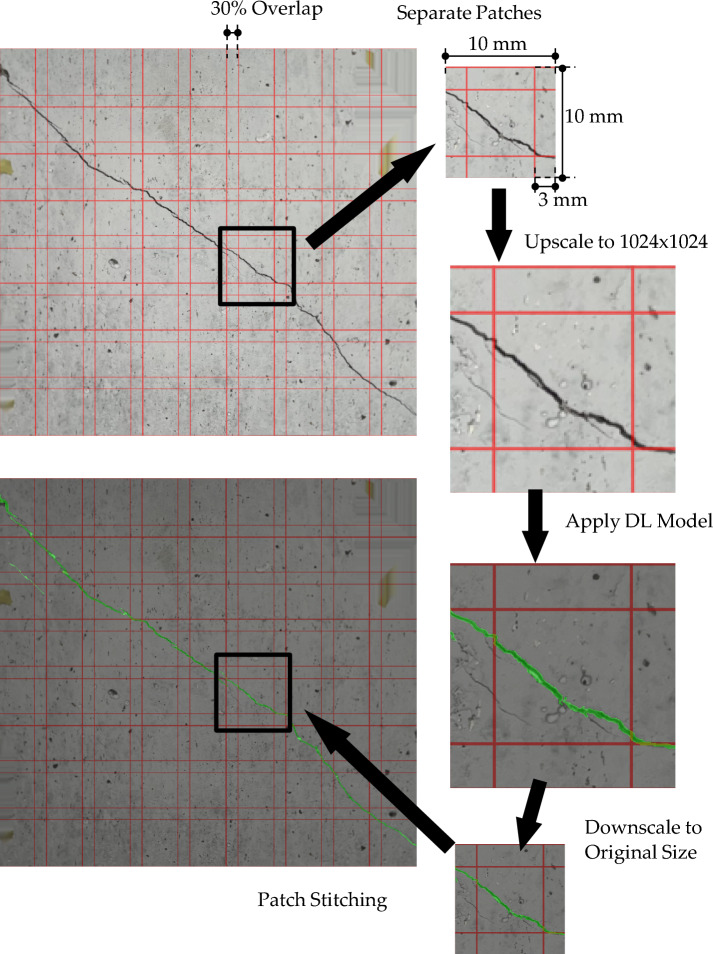
Framework of crack detection and segmentation.

For crack width estimation, crack segmentation is performed by applying the built DL model to crack images. As depicted in Fig. 4, the input image is divided into overlapping square patches with an overlap of 30%. The DL model processes each patch to generate per-pixel confidence masks. Next, these masks are stitched to produce a full-resolution confidence map for the entire image, from which a binary crack mask is derived. The crack skeleton is then extracted and refined. Finally, local crack widths are quantified using metric maps informed by the image scale, which is obtained via checkerboard-based homography. The details of each step of the process of crack width measurement will be discussed in the following sub-sections.

### Patch grid with overlap

The image is divided into patches of size 10 mm/patch with an overlap of 30%. Next, patches are extracted in pixel units and upscaled to size 1024 × 1024. Next, these patches are processed by the built segmentation DL model to produce a crack confidence map per patch. The efficiency of this process is tracked via object confidence (certainty level of crack existence) and pixel mask probability (probability of each pixel within the crack). Finally, a per-pixel patch probability is computed in Eq. (9) by multiplying pixel mask probability at location (i, j) by object confidence and selecting per-pixel maximum over instances^63^.

9 $$p_{patch} \left( {i,j} \right) = \mathop {\max }\limits_{k \in instances} \left( {p_{k} \left( {i,j} \right) \cdot c_{k} } \right)$$

where, *p*_*k*_ is the model mask probability for instance *k*, and *c*_*k*_ is instance confidence.

### Stitching per-patch maps

Each patch produces a probability map, where each pixel has a crack probability ranging from 0 to 1. Due to the overlap of the patches, pixels in the full image may appear in multiple patches. To decide the final crack probability for each pixel in the full image, the maximum among patch probabilities is considered. This constructs a full-image confidence map *p*_*full*_* (x,y)* using Eq. (10):

10 $$p_{full} \left( {x,y} \right) = \mathop {\max }\limits_{{r:\left( {x,y} \right) \in patch_{r} }} p_{patch,r} \left( {x^{\prime},y^{\prime}} \right)$$

where, r is the patch index, *p*_*full*_* (x,y)* is the final crack probability assigned to pixel *(x,y)*, *p*_*patch,r*_ (*x*′,*y*′) is the predicted crack probability at location (*x*′,*y*′) in the local coordinate system of each patch.

### Skeletonization and mask refinement

For each detected crack, the Euclidean distance transform *D(x,y)* of the binary foreground mask is calculated in Eq. (11). Next, the skeleton is computed by thinning the binary mask into a 1-pixel-wide medial axis using an iterative thinning^64,65^.

11 $$D\left( {x,y} \right) = \mathop {\min }\limits_{q \in B} \left( {x,y} \right) - q$$

where, *B* is the background set (zeros), and *q* is the spatial coordinate belonging to the set *B*.

To refine detected crack boundaries and correct small localization errors, an energy-minimization approach (GrabCut) is implemented^66^. In this approach, the image is represented as graph, where each pixel is modelled as a node connected to its neighbors as well as two special nodes corresponding to foreground and background. Refinement is performed by computing the minimum cut of this graph, which yields the optimal separation between foreground and background regions. This cut minimizes an energy function, so called Markov Random Field (MRF)^67,68^. The refining process starts by seeding the GrabCut model from the crack skeleton, which serves as definite foreground. Two probability thresholds $p_{lo} \left( {0.03} \right)$ and $p_{hi}$ (0.55) are applied to classify pixels as likely foreground or background during the iterative process. The classification of pixels is as follows: pixels corresponding to the component skeleton (definite foreground), pixels with model confidence $p \ge p_{hi}$. (probable foreground), pixels with model confidence $p \le p_{lo}$ (definite background), and remaining pixels (probable background).

#### Local width estimation

Using the refined mask of the crack skeleton, the crack width is calculated using the gradients of the distance transform method (DTM)^69,70^. DTM is described as contour lines extending from the crack skeleton towards the crack edge. The contour lines range from zero at the crack edge to maximum distance *d* at the skeleton. Subsequently, at each pixel on the crack skeleton, the local normal direction of crack width is estimated by taking the gradient of that pixel computed in Eq. (12). Finally, the crack width of each pixel on the crack skeleton is computed in Eq. (13).

12 $$g = \nabla {\mathrm{D}}\left( {{\mathrm{x}},{\mathrm{y}}} \right) \approx \left( {\frac{1}{2}\left( {D\left( {x + 1,y} \right) - D\left( {x - 1,y} \right)} \right),\frac{1}{2}\left( {D\left( {x,y + 1} \right) - D\left( {x,y - 1} \right)} \right)} \right)$$

13 $$w_{mm} \left( {x,y} \right) = 2.r\left( {x,y} \right).\alpha$$

where, *g* is the gradient vector of the distance transform at pixel (x,y), *D(x,y)* is the EDT value at spatial coordinate *(x,y)*, and *r(x,y)* is the local crack radius at the skeleton point *(x,y)*, which equals *D(x,y)* as the distance transform returns the minimum distance to the boundary (half of the crack width in pixel units).

## Results and validation

This section presents the validation results of the proposed framework for crack detection and crack width quantification. The DL segmentation model was evaluated using standard performance metrics to assess its detection capabilities. In parallel, the crack width measurement was validated using an independent dataset obtained from large-scale experimental specimens and existing RC structures.

### Deep learning model

The final trained dataset went through several refinement stages to achieve the best results for validation of the proposed DL model and to enhance its ability to detect surface cracks in various environmental conditions. The effect of adding artificial lines to the trained dataset is tested, as well as the effect of local enhancement. Eventually, the performance and outcomes of the optimized DL model are presented and discussed.

### Effect of adding artificial lines

**Fig. 5:**
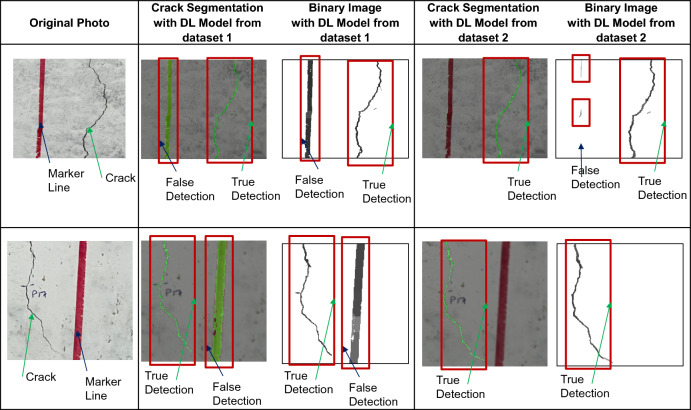
Examples of enhanced performance of the DL model after adding artificial lines to the training dataset.

As mentioned earlier, artificial lines were added to the dataset so that the DL model is able to differentiate between real cracks and other markers/signs on the concrete surfaces. Figure 5 shows an example when the DL model is trained using dataset 1 and dataset 2 (with and without artificial lines, respectively). The performance of crack detection has been significantly improved, as the model with dataset 1 falsely detected red marker lines as cracks. In contrast, the other model was able to distinguish between marker lines and concrete surface cracks. Thus, it was decided to build the DL model using the dataset with artificial lines.

### Effect of local enhancement

In order to demonstrate the impact of local enhancement, the DL model is trained using dataset 3 and dataset 4 (with and without applying local enhancement, respectively). To assess the performance of the built DL models, detection and segmentation metrics are used, including precision, recall, F1 score, and mAP@0.5. Precision defines the accuracy of detected cracks, while recall ensures that all cracks located in a single photo are detected. F1 score combines both precision and recall in one metric, as well as mAP@0.5. The formulas of these metrics are defined in Eqs. (14) to (17).

As shown in Table 1, the application of local image enhancement leads to a remarkable improvement in the performance metrics of the trained model. Overall, the metrics improved by 7%-19% across the evaluated metrics of detection and segmentation. The detection metrics evaluate the model’s capability to correctly identify the presence of cracks within the images, whereas the segmentation metrics assess its accuracy in precisely detecting crack boundaries and edges. For crack detection, precision increases by 8.7%, which indicates a reduction in false-positive predictions, while recall improves by 7.0%, which demonstrates an enhanced ability to identify true crack instances. Additionally, the mAP@0.5 metric increases by 12.2%, which reflects improved overall detection accuracy across confidence thresholds, and the F1 score is enhanced by 8.6%, confirming improved balance between precision and recall. In the case of crack segmentation, other remarkable improvements are demonstrated. Precision increases by 13.9%, recall by 11.5%, mAP@0.5 by 19.0%, and F1 score by 13.6%. These improvements indicate that the model is more effective in capturing crack geometry and boundaries after local enhancement.

14 $$P = \frac{TP}{{TP + FP}}$$

15 $$R = \frac{TP}{{TP + FN}}$$

16 $$mAP = \frac{{\mathop \sum \nolimits_{i = 1}^{K} AP_{i} }}{K}$$

17 $$F1 = \frac{2 \times P \times R}{{P + R}}$$

where, precision (*P*), recall ®, mean average precision (*mAP*), and *F*1 score are used to evaluate the performance of the trained model, *TP* is true-positive, *FP* is false-positive, *FN* is false-negative, and *K* is the number of classes. *AP* is the area surrounded by the *P-R* curve, which can be expressed in Eq. (18), and *mAP* is the mean of *AP* values for all classes – see Eq. (18).

18 $$AP = \mathop \smallint \limits_{0}^{1} P\left( r \right)dr$$

**Table 1:** Validation results of DL models trained using dataset 3 and dataset 4.

Parameter	Detection of DL model using dataset 3	Detection of DL model using dataset 4	Segmentation of DL model using dataset 3	Segmentation of DL model using dataset 4
Dataset size	9681	19,362	9681	19,362
Precision (P) (%)	84.90	92.30	69.30	78.90
Recall (R) (%)	78.50	84.00	64.10	71.50
mAP@0.5	0.82	0.92	0.58	0.69
F1 Score	0.81	0.88	0.66	0.75

### Proposed DL model results

**Fig. 6:**
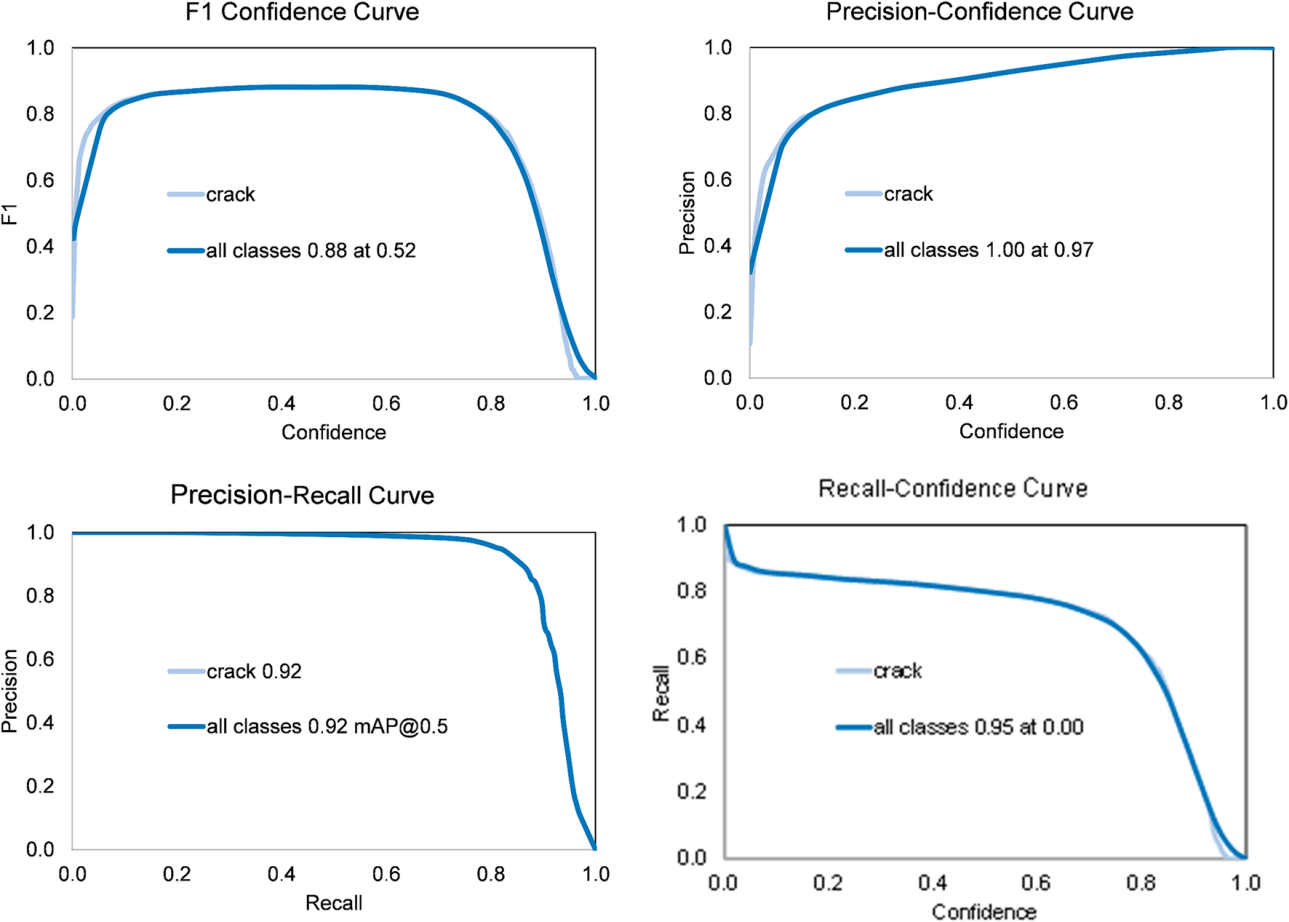
Performance metrics of crack detection of the proposed DL model.

**Fig. 7:**
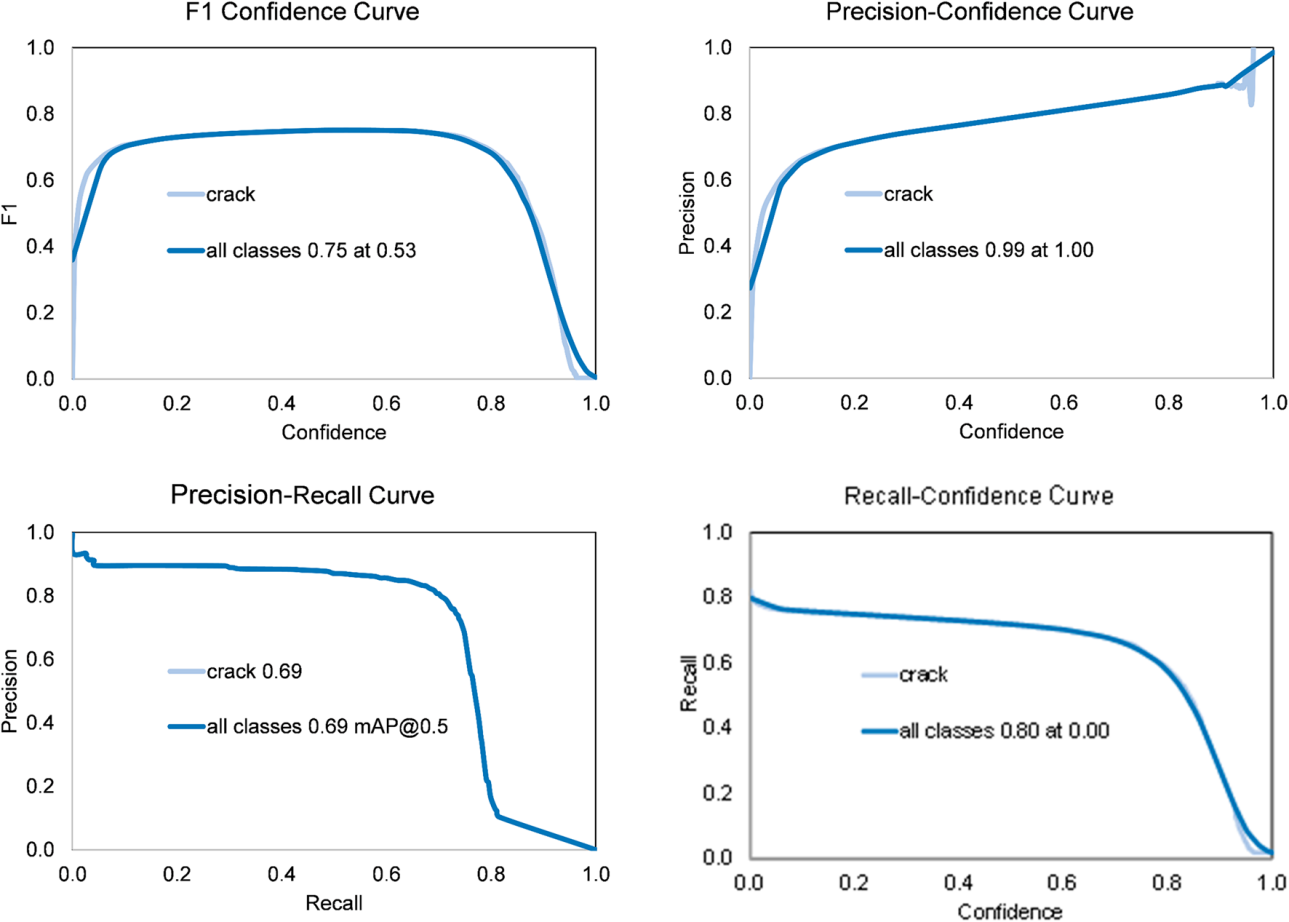
Performance metrics of crack segmentation of the proposed DL model.

As discussed earlier, adding artificial lines to the trained dataset and applying local enhancement can significantly improve the performance of the trained DL model. Consequently, the final DL model is trained using dataset 4 after applying the above-mentioned techniques. Figure 6 and Fig. 7 illustrate the post-training performance of the proposed model for crack detection and crack segmentation, respectively. The observed performance improvements are expected due to the role of local image enhancement in increasing crack visibility and contrast relative to the surrounding background. By emphasizing crack features and suppressing irrelevant background variations, the local enhancement process facilitates feature extraction and enables the DL model to focus more effectively on crack regions. Consequently, the model shows improved localization, reduced ambiguity in predictions, and greater reliability in botdetection and segmentation of concrete cracks.

**Fig. 8:**
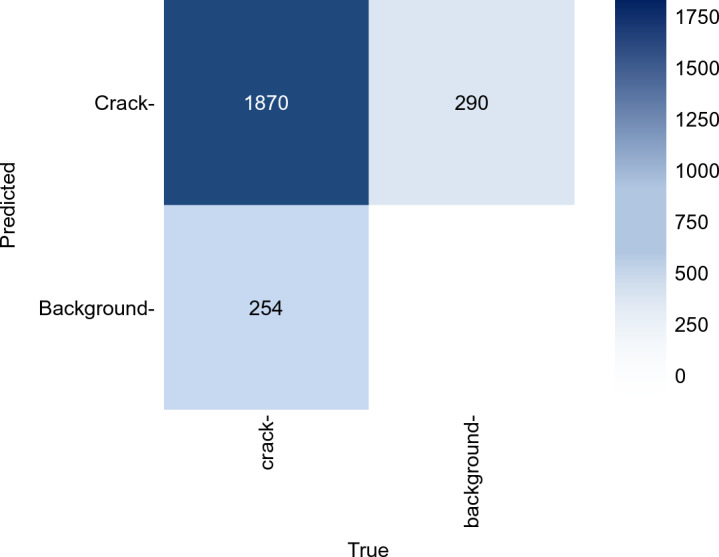
Confusion matrix of the test dataset.

**Fig. 9:**
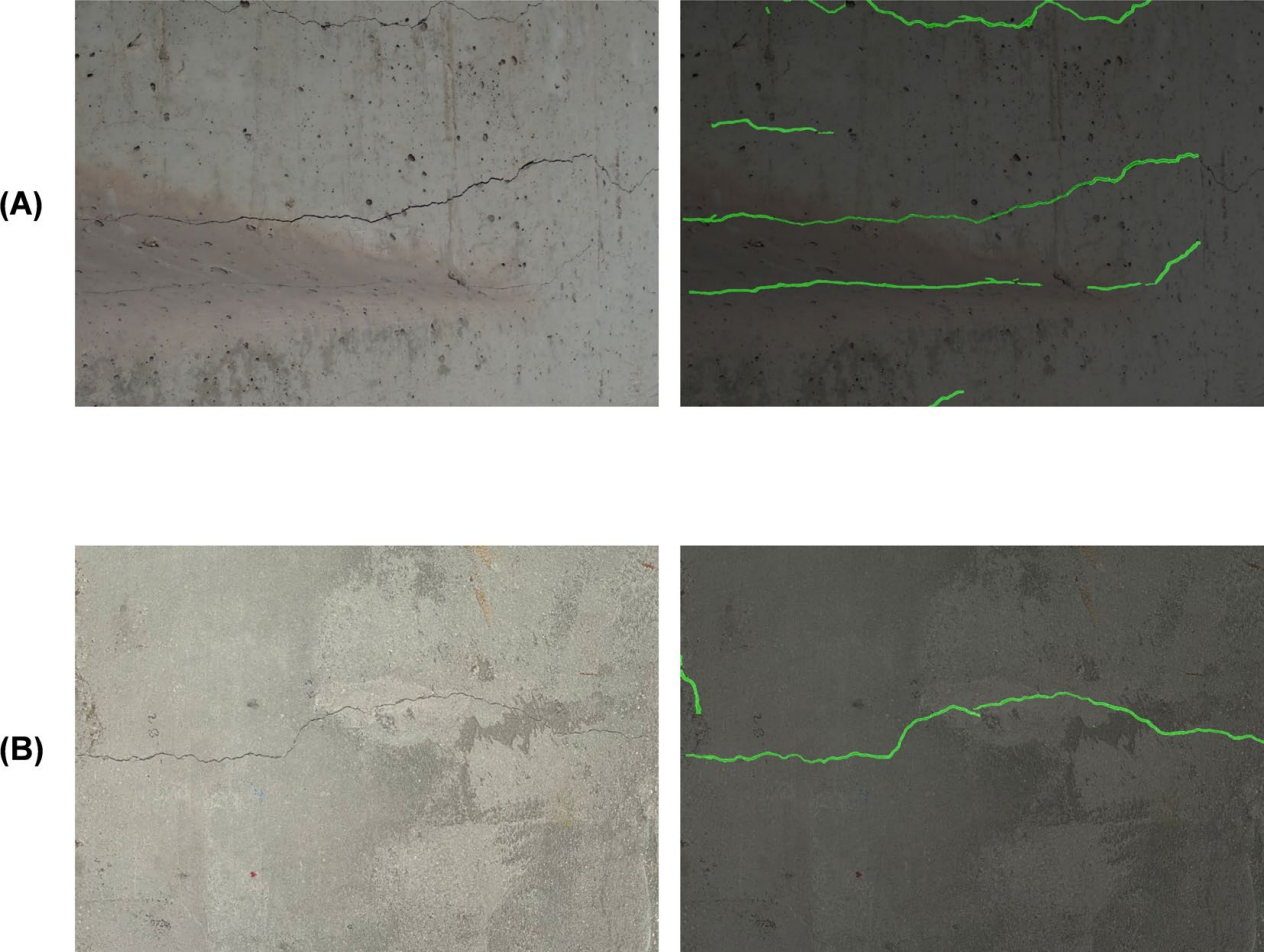
Examples of cracks detection in existing structures. (**A**) RC girder in existing bridge, and (**B**) RC slab in existing building

The performance of the DL model on the validation and test subsets is illustrated in the confusion matrix shown in Fig. 8. The confusion matrix summarizes the model’s classification accuracy by reporting the percentages of true-positive, false-positive, and false-negative. The results are calculated based on Intersection over Union (IOU) equals to 0.8, which is a relationship between the predicted boundaries generated by the DL model and the ground truth. Under this criterion, the model correctly identifies 83% of true crack instances (true-positives), while the false-negative rates correspond to 17% of the true instances. Finally, the built DL model is applied in real scenarios to detect cracks in existing structures. Figure 9 shows examples of detecting cracks in existing RC structures (i.e., an RC girder in in-service bridge and an RC slab in an existing building).

### Crack width measurements

**Fig. 10:**
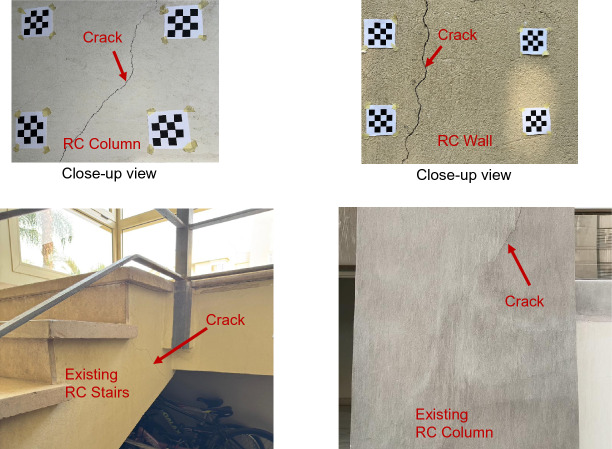
Samples of RC cracks captured from existing RC buildings.

**Fig. 11:**
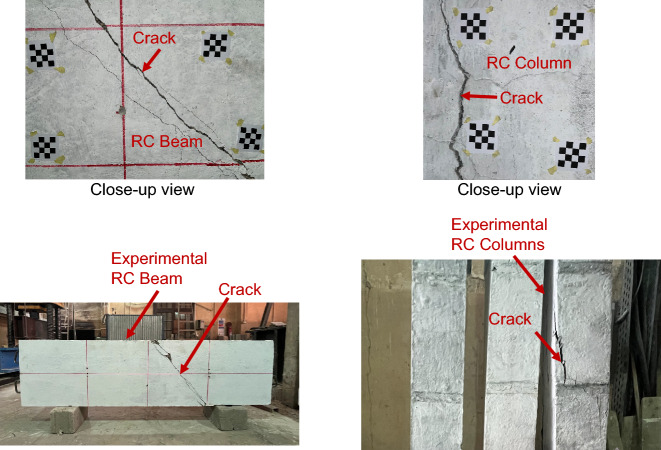
Samples of RC cracks captured from experimental specimens.

**Fig. 12:**
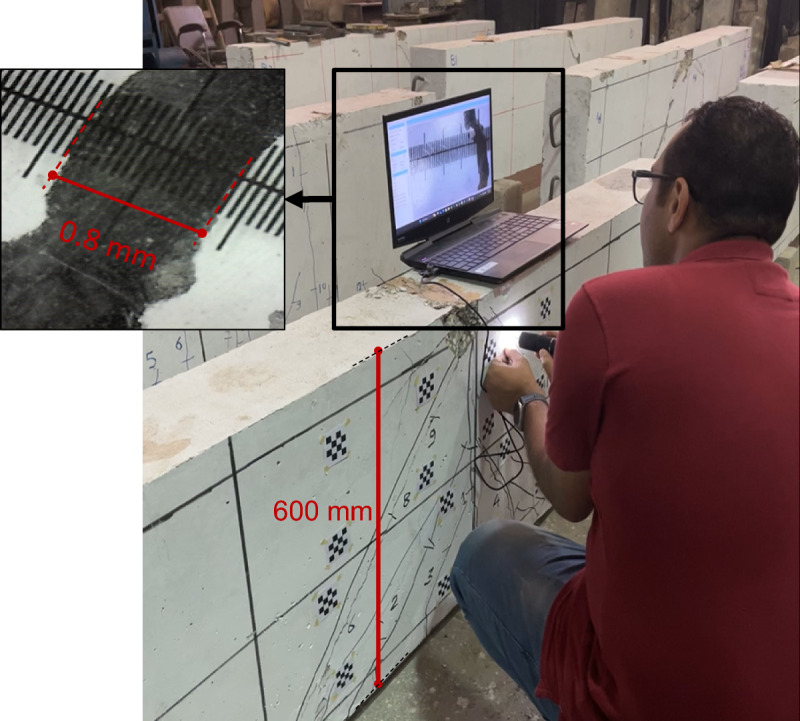
Validation and data collection of crack width measurements using a digital microscope and a crack comparator.

To validate the proposed framework in terms of crack width measurement, a validation dataset was collected based on precise manual measurements. In the real world, visibility of cracks is affected by a wide range of uncontrolled factors, such as variation in lighting conditions, surface roughness, staining, aging effects, and possible concrete spalling. Thus, the inclusion of real-structure data was necessary to secure the reliability of the proposed method. The validation dataset includes a total of 230 sample crack points obtained from different types of RC members in existing buildings (67 Samples) – see examples in Fig. 10 and tested experimental specimens (163 samples) – see examples in Fig. 11. Manual measurements of crack widths were carried out using a high-resolution digital microscope attached with a crack comparator with an accuracy of 0.05 mm, as shown in Fig. 12. The measured crack widths range from 0.1 mm to 1.8 mm for existing RC buildings and from 0.1 mm to 3.2 mm for experimental specimens.

**Fig. 13:**
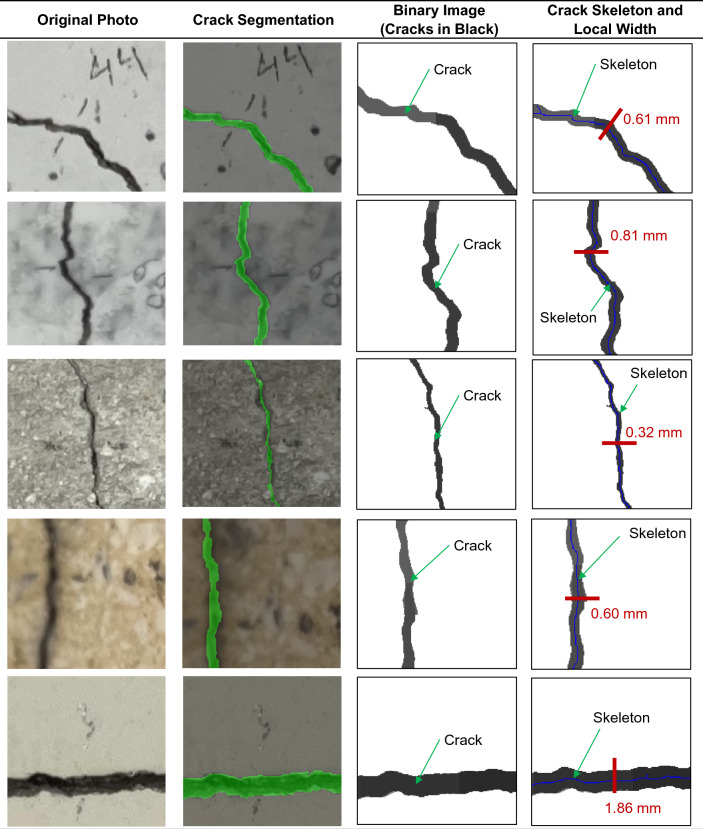
Examples of the process of predicting crack widths using the proposed framework.

**Fig. 14:**
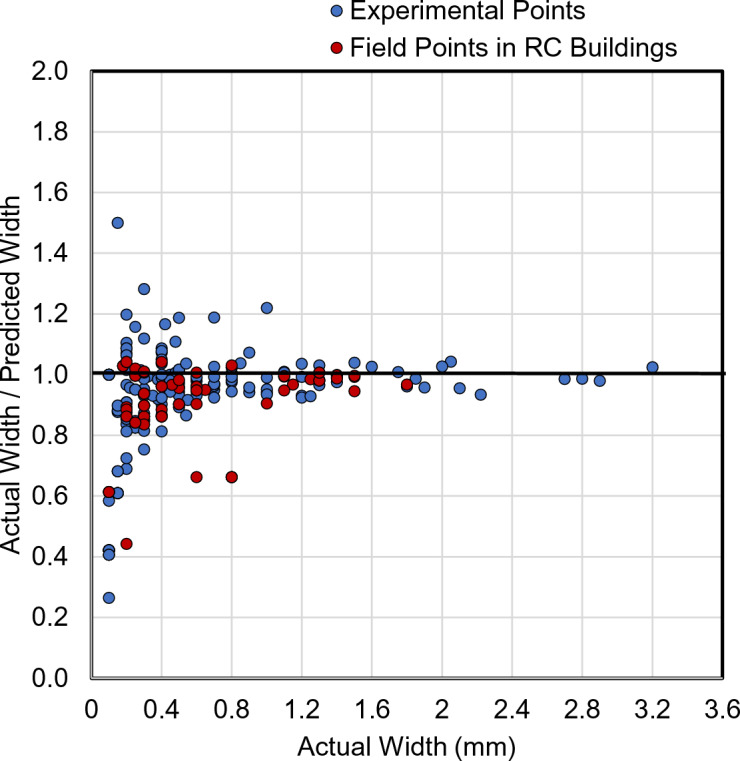
Validation of the proposed framework in terms of crack width measurement.

In parallel with the manual measurements, digital images were captured for each crack location corresponding to the selected sample points in the validation dataset. These images were subsequently processed using the proposed framework to automatically detect cracks and estimate their corresponding widths. Examples of crack detection results and crack width predictions are presented in Fig. 13, demonstrating the method’s capability to identify crack locations and quantify crack widths simultaneously. Figure 14 presents the validation results in terms of actual-to-predicted-crack-width ratios, producing an average of 0.93 and a coefficient of variation (COV) of 16.82%. In addition, the mean absolute error (MAE) of the predicted crack widths is 0.04 mm, while the mean relative error (MRE) is 12.65%. Collectively, these results demonstrate the reliability of the proposed framework. Furthermore, from a structural assessment perspective, the method provides a conservative evaluation by slightly overestimating crack widths, thereby securing the safety of structural performance assessments.

It is also important to evaluate the performance of the proposed framework separately for field data and experimental data. For the field points, the COV is 9.87%, with a mean value of 0.94. The MAE of the predicted crack widths is 0.03 mm, corresponding to MRE of 8.20%. For the experimental points, the COV is 19.03%, with an average value of 0.93. The MAE of the predicted crack widths is 0.04 mm, with an MRE of 14.42%. These results indicate that the proposed framework confirms generalization capability, as it yields consistent with unbiased predictions across both field and experimental datasets.

For further investigation of the validation results, the validation dataset was divided into two distinct groups according to the magnitude of crack width. Group 1 for cracks wider than or equal to 0.50 mm, containing 102 crack points, while Group 2 for cracks narrower than 0.50 mm, containing 128 crack points. In the case of Group 1, the COV for the actual-to-predicted crack-width ratios is 5.77%, with an average of 0.98. On the other hand, the statistics of Group 2 of cracks result in a COV of 21.86%, with an average of 0.89. According to these results, it is clear that the proposed method performs better for wider crack with less conservatism. These results were expected as the DL model can accurately identify the crack edges of wider cracks at a higher confidence level. In other words, the pixel-size was sufficient to capture the crack width of wider cracks. To increase the accuracy of the prediction of thinner cracks, employing higher-resolution imaging systems or closer-range image acquisition can enhance the results.

### Application and future perspective

**Fig. 15:**
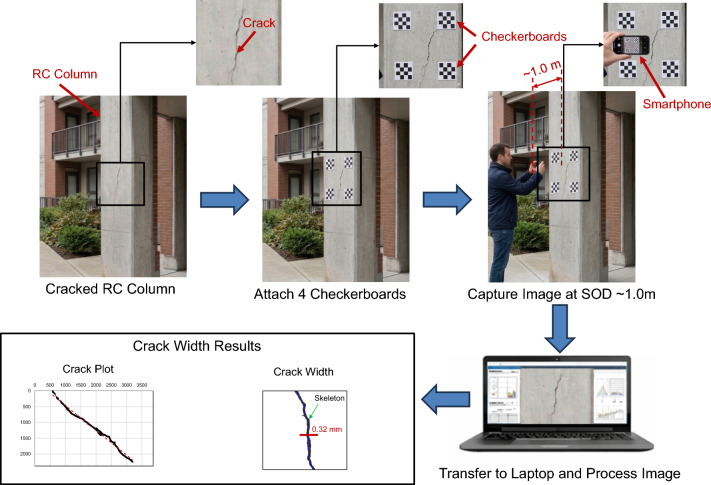
Implementation procedures of the proposed method.

The proposed framework introduces a low-cost approach for crack detection and crack width measurement, utilizing widely accessible devices such as smartphones. The applicability of the method has been successfully validated in real scenarios (i.e., slabs, beams, and columns in existing RC buildings). Figure 10 shows an example of an RC residential building exhibiting cracks due to differential foundation settlement. The implementation procedure of the proposed method is summarized in Fig. 15. First, calibration boards are temporarily attached to the concrete surface. Next, images are captured using a smartphone camera (12-megapixel resolution) from a stand-off distance (SOD) of approximately 1.0 m. Based on the camera specifications, this distance corresponds to a field of view of approximately 400 mm × 300 mm. The captured images are then transferred to a laptop for processing using the developed software. Finally, the software performs post-processing on the images, providing crack coordinates along with corresponding crack width measurements.

Generally, the results of the proposed framework demonstrate a satisfactory level of accuracy alongside the potential for further refinement and enhancement. Despite these promising outcomes, certain limitations remain. In particular, the current dependence on calibration checkerboards, together with the restriction imposed by relatively short SOD, constrain the practical applicability of the method in some scenarios. This is evident in the case of RC members that are not directly accessible without specialized access equipment, such as bridge girders and the bottom side of bridge decks. The above-mentioned limitations can be addressed through emerging technological advancements. For instance, the integration of LiDAR systems, which are increasingly available in modern smartphones, offers a viable alternative for camera calibration without the need of physical checkerboards. Such capabilities could significantly enhance the flexibility and field applicability of the framework. Furthermore, SOD can be extended using higher-resolution cameras.

**Fig. 16:**
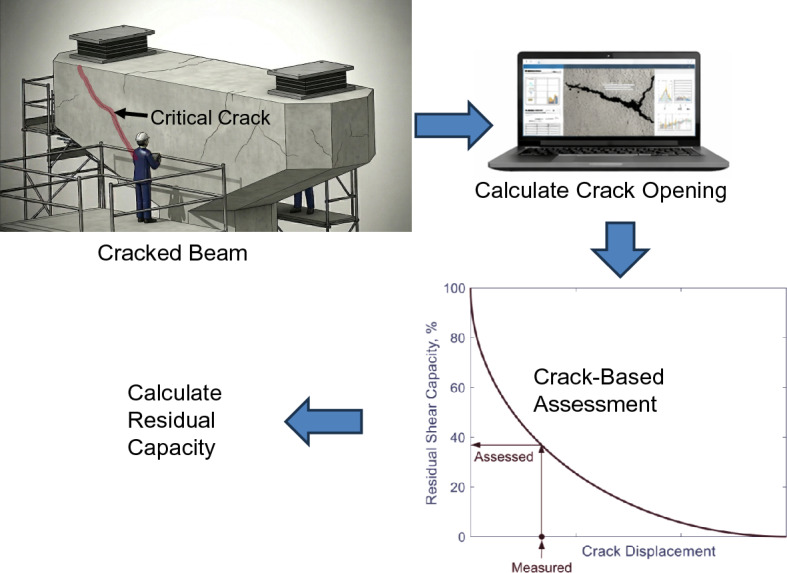
Future perspective of crack-based assessment of RC structures.

Beyond the scope of this paper, the proposed framework can be extended to differentiate between various crack types (e.g., shear and flexural) based on crack orientation, as crack angles can be directly derived from the computed crack coordinates. Furthermore, the framework can be expanded to conduct structural assessments of existing RC members. Given the structural element type (beam, column, shear wall, etc.), established crack-based assessment (CBA) methods reported in the literature^6–9^ can be readily applied to evaluate the residual capacity of RC elements – see Fig. 16. In the scheme of CBA methods, they rely on crack geometry and the magnitude of crack opening, which can be obtained from the proposed framework.

## Conclusions

This research proposes a comprehensive and practical framework for automated crack detection and crack width measurements in RC structures. The proposed framework is based mainly on a DL model for crack detection and segmentation, which was built using thousands of crack photos. For crack width measurements, a post-processing procedure incorporating a calibration method was implemented. The framework was validated using crack measurements obtained from both experimental specimens and existing RC structures, ensuring its applicability in real-world conditions. According to the proposed framework and validation results, the following conclusions can be drawn:

- The proposed framework is capable of accurately detecting cracks and measuring their widths in various types of RC structures using low-cost and readily available tools, such as smartphone cameras and checkerboard-based calibration, without requiring skilled operators or specialized inspection equipment. This highlights its suitability for rapid low-cost inspections.
- The deep learning model demonstrates high performance in crack detection and segmentation, as reflected by performance metrics (e.g., F1 score of 0.88), confirming its reliability in identifying cracks.
- In real inspection scenarios, concrete surfaces may include thin non-crack features such as old inspection markers. Augmenting the training dataset with artificial line patterns significantly enhances the capability of the DL model, reducing false detections and improving performance in realistic field conditions.
- The crack width measurement of the framework was validated using 230 manually measured sample points. For cracks wider than or equal to 0.50 mm, the method achieved high accuracy, with a coefficient of variation (COV) of 5.77% with an average actual-to-predicted ratios of 0.98. For cracks narrower than 0.50 mm, the variability increased (COV = 21.86%, average = 0.89), reflecting the challenges associated with pixel resolution and boundary detection for very thin cracks.
- From an assessment perspective of existing RC structures, the framework is conservative, as it tends to slightly overestimate crack widths. This conservatism is beneficial and enhances the reliability of structural safety evaluations.
- The proposed framework establishes a solid foundation for a generalized and robust crack detection methodology for RC structures for short SOD (1.0 m). Future enhancements may focus on using higher-resolution cameras for extending SOD and expanding the training dataset to include a broader range of crack images captured from diverse RC structures and varying environmental conditions, thereby further improving the model’s generalization and reliability. Additionally, the calibration process for crack width measurement can be automated by using modern mobile devices. Recent smartphones equipped with LiDAR systems offer promising capabilities for accurate calibration, enabling more efficient on-site implementation of the proposed framework.

## Data Availability

Data sets generated during the current study are available from the corresponding author on reasonable request.
